# Multi-Source Pansharpening of Island Sea Areas Based on Hybrid-Scale Regression Optimization

**DOI:** 10.3390/s25113530

**Published:** 2025-06-04

**Authors:** Dongyang Fu, Jin Ma, Bei Liu, Yan Zhu

**Affiliations:** 1 School of Electronics and Information Engineering, Guangdong Ocean University, Zhanjiang 524088, China; 2 Guangdong Provincial Marine Remote Sensing and Information Technology Engineering Technology Center, Zhanjiang 524088, China

**Keywords:** pansharpening, sentinel remote sensing imagery, mixed-scale regression, mutual information, South Sea Islands

## Abstract

To address the demand for high spatial resolution data in the water color inversion task of multispectral satellite images in island sea areas, a feasible solution is to process through multi-source remote sensing data fusion methods. However, the inherent biases among multi-source sensors and the spectral distortion caused by the dynamic changes of water bodies in island sea areas restrict the fusion accuracy, necessitating more precise fusion solutions. Therefore, this paper proposes a pansharpening method based on Hybrid-Scale Mutual Information (HSMI). This method effectively enhances the accuracy and consistency of panchromatic sharpening results by integrating mixed-scale information into scale regression. Secondly, it introduces mutual information to quantify the spatial–spectral correlation among multi-source data to balance the fusion representation under mixed scales. Finally, the performance of various popular pansharpening methods was compared and analyzed using the coupled datasets of Sentinel-2 and Sentinel-3 in typical island and reef waters of the South China Sea. The results show that HSMI can enhance the spatial details and edge clarity of islands while better preserving the spectral characteristics of the surrounding sea areas.

## 1. Introduction

With the advancement of image acquisition technologies, high-precision satellite images play a vital role in various important marine remote sensing applications, including environmental monitoring [1,2], spectral decomposition [3,4], water quality assessment [5,6], and water color retrieval [7,8,9]. However, acquiring satellite remote sensing images with high spatial and spectral attributes through a single sensor is often a challenging task due to the limitations of optical remote sensing sensors [10]. For this reason, multi-source fusion technology has emerged, capable of generating high-quality remote sensing images with high spatial resolution and high spectral information by integrating remote sensing data from different sensors with different resolutions and spectral ranges.

At the same time, many existing Earth observation programs have attempted to alleviate these limitations by incorporating multiple dedicated satellites to meet specific spatial and spectral requirements. The Sentinel-2 (S2) and Sentinel-3 (S3) satellites are examples of this trend. The S2 satellite [11] carries a Multispectral Imager (MSI) sensor providing 13 spectral bands in the 443–2190 nm wavelength range with a spatial resolution of 10 to 60 m, while the Sentinel-3B (or Sentinel-3A) satellites [12] both carry the Ocean and Land Colour Imager (OLCI). The image produced by the OLCI consists of 21 spectral channels ranging from about 400 nm to 1020 nm, while the OLCL product, with its limited coarse spatial resolution of 300 m, is more focused on spectral features of the oceans, inland waterways, and coastal areas [13]. However, this also limits the application of OLCI imagery at local scales, particularly for heterogeneous landscape features.

In applying localized scenes of island waters in this paper, to improve the spatial resolution of the Sentinel-3 OLCI images, they can be fused with the spatial detail information provided by the Sentinel-2 MSI. It should be noted that marine image fusion is a different process from terrestrial image fusion because the intrinsic characteristics of water bodies in island waters are constantly changing. The two images to be fused will have non-negligible differences in spectral and spatial characteristics, and inconsistent acquisition times can lead to spatial blurring and spectral distortion. This problem cannot be solved by geographically weighted regression point-to-surface information fusion methods. Therefore, we tend to use covariance-guided feature-matching methods for spatial–spectral information fusion to alleviate this problem. At the same time, the unprecedented cross-sensor sentinel data also provides us with the opportunity to address these limitations using pansharpening methods within the framework of spatial–spectral information fusion from the perspective of injection-gain-based processing.

Classical algorithms for pansharpening continue to occupy a significant position in the field, offering a notable advantage in high computational efficiency. These algorithms can be broadly classified into three main categories [14], as outlined in the following section. These include component substitution (CS), multi-resolution analysis (MRA), and variational optimization (VO)-based methods. The initial approach, frequently designated as the ‘spectral class’, entails projecting the original MS image into the transform domain to isolate its spatial information and replace it with a PAN image. A considerable number of pioneering pansharpening techniques are classified as belonging to the CS class, largely due to the ease with which they can be implemented. Notable examples include Intensity–Hue–Saturation (IHS) [15], Principal Component Analysis (PCA) [16], Gram–Schmidt (GS) spectral sharpening, GS adaptive methods [17], and Band-Dependent Spatial Detail (BDSD) [18]. The latter are frequently designated as “spatial classes”, these include the additive wavelet luminance proportional (AWLP) [19], morphological filters (MFs) [20], generalized Laplace pyramid (GLP) [21], Morphological Pyramid [22], and Frame Boosting [23]. In comparison to component substitution class fusion methods, multi-resolution analytical fusion methods typically demonstrate superior spectral fidelity and are relatively unaffected by radiometric differences between multi-source images.

In recent years, VO methods have gained popularity due to the development of convex optimization and inverse problems. These methods employ observation models and sparse representations to construct an energy function, which is then solved using an optimization algorithm to obtain a pansharpening image. The most commonly used VO-based methods are P+XS, regularized solution of the inverse problem, coupled non-negative matrix factorization (CNMF) [24], and sparse representation. However, the computational cost is relatively high, as the target image is usually estimated under the assumption of properly co-aligned PAN and LRMS images. In contrast to traditional methodologies, deep learning-based approaches (DL), which directly learn the mapping from observed actual values to real values through neural networks, have emerged as a prominent area of interest in recent years. Prominent examples include PNN [25] and PANnet [26]. Most of these deep learning-based methods rely on training with large datasets, yet this is constrained by asynchronous data types from different satellites and the scarcity of remote sensing datasets in marine domains. (This manifests as a scarcity of high-quality paired training data in cloudy island marine environments, often accompanied by temporal mismatches.) Furthermore, training deep neural network models requires significant time investment and poses challenges in optimizing optimal parameters.

Notwithstanding the favorable outcomes of the aforementioned and associated methodologies, certain challenges persist in the context of highly heterogeneous sensors (e.g., S2/S3) and oceanic island scenes. To illustrate, the texture features derived from OLCI products invariably exhibit some degree of spatial distortion, which may prove detrimental to the resulting pansharpening outcomes. Furthermore, the local water scenes of islands present significant spectral loss issues that require a more precise solution to be addressed concurrently.

**Figure 1:**
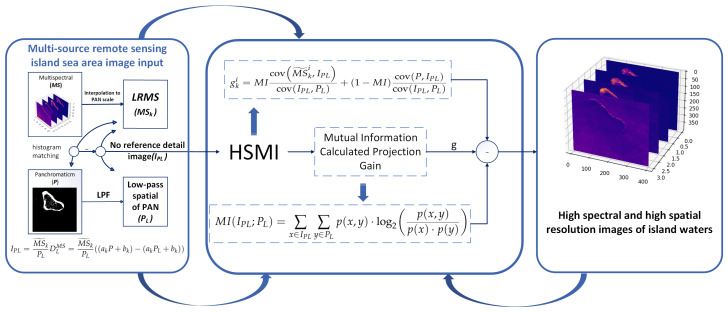
MTF-GLP-HPM-HSMI flowchart.

The MRA method, on the other hand, does not need to provide training data and is more effective than CS in preserving the spectral features of the original MS images. Given the above problems, in this paper, the MRA method based on scale regression is proposed for the improvement of low spectral retention of island waters with large resolution differences between multi-source sensors, i.e., MTF-GLP-HPM-HSMI based on mutual information hybrid-scale regression method. The hybrid-scale information, including the detailed image with spatial residuals as well as the spectral residuals, is combined and fitted by the mutual information to compute the hybrid weighting coefficients to obtain the final pansharpening results. Experimental results show that our proposed MTF-GLP-HPM-HSMI method outperforms various popular MRA methods in island sea application scenarios. The technical route of this paper is shown in Figure 1.

Briefly, the main contributions of this work can be summarized as follows: 1. To address the problem of spectral aberrations in water bodies in island waters, we propose a novel MRA pansharpening method (MTF-GLP-HPM-HSMI), which incorporates mixed-scale information (including detailed images with spatial and spectral residuals) into scale regression to generate more accurate pansharpening results. 2. By introducing mutual information to calculate the hybrid weighting coefficients, the intrinsic relationship between spatial and spectral information can be more accurately represented, achieving a more balanced fusion representation across different scales. 3. When fusing S3-OLCI and S2-MSI data, the performance of several popular pansharpening methods is compared and analyzed, and HSMI can better balance the enhancement of island spatial details and edge clarity with the retention of the spectra of the sea area around the island.

## 2. Related Work

### 2.1. Inject Gain

Several algorithms have been developed for the fusion of multi-source satellite remote sensing data [27]. However, the majority of these algorithms are designed for the fusion of Landsat-8 and Sentinel-2 imagery, as well as the enhancement of auxiliary images using Sentinel satellite data [28]. For instance, Wang et al. [29] extended the aspect regression kriging method (ATPRK) to Landsat-8 and Sentinel-2 image fusion, employing the high spatial resolution bands in both images as an auxiliary reference. Similarly, Shao et al. [30] devised a super-resolution network (ESRCNN) with the specific purpose of fusing multiple sources of remote sensing data in order to obtain coordinated remote sensing reflectance products. Additionally, pansharpening represents a category of techniques within the aforementioned image fusion paradigm [31]. It is undeniable that unsupervised deep learning-based pansharpening methods have achieved significant progress in the field of multi-source information fusion. However, it is disappointing that the fusion performance of these algorithms is suboptimal in specific island water scenarios. According to analysis, the spatial resolution of Sentinel-3 OLCI is relatively low, and the spatial information available from Sentinel-3 OLCI multispectral images in island scenarios is limited (islands are small in area, and the limited resolution of satellite remote sensing results in fewer learnable pixels in the input images, with sparse spatial details). This restricts the ability of deep networks to learn meaningful spatial–spectral relationships, especially in dynamic marine environments where water bodies exhibit rapid spectral variations. In this context, we have chosen an MRA-based injection gain framework for multi-source fusion while prioritizing the preservation of the spectral characteristics of the original MS images.

Two widely accepted models for injection gain in MRA modeling are high-pass modulation (HPM) [32] and context-based decision-making (CBD) [33]. In [34], a model based on full-size (FS) regression is proposed that employs HRMS and PAN images instead of their low-resolution (LR) degraded versions for estimation. Furthermore, in [35], a model grounded in dual-scale (DS) regression was introduced, wherein the injection gain is computed through a linear combination of high-resolution (HR) and low-resolution (LR) projection coefficients. This model is regarded as an extension of the regression-based HPM model, and the MTF-GLP-HPM-R [36] model, which is based on multiple linear regression. However, the majority of regression-based methodologies are constrained by the unavailability of high-resolution (HR) images [34]. This degradation inevitably results in a considerable loss of information, particularly in images with intricate details. This paper aims to provide insights into mixed-scale regression methods that inject detail gain into HPM models. Firstly, the information in high-resolution data (e.g., PAN images or their details) can be fully utilized to improve the accuracy of the estimation. Secondly, the residual information between the detail images and the (HR and LR) images is taken into account, and the injection weights are calculated using correlation coefficients to preserve the spectral details better.

### 2.2. Ocean Color Inversion in Island Waters

Satellite remote sensing has significantly propelled the progress of ocean color research. However, in the context of island waters, as discussed in this paper, the optical properties of near-island waters are complex, and their optical characteristics change rapidly. Therefore, such near-island water inversion requires more spectral information, including more spectral channels and higher spectral resolution, while also needing to enhance high-resolution texture spatial features as much as possible. Additionally, factors such as different satellite sensors and cloud interference affect the accuracy of data obtained by satellite sensors, thereby limiting effective sampling of the ocean [37,38]. Currently, popular multi-source remote sensing data fusion methods for water color inversion typically rely on the interpolation and matching of water color products, which limits their application to single products. Meanwhile, methods primarily based on multi-temporal data fusion are mostly suitable for the fusion of inland water bodies [39,40].

To address these challenges, this study takes raw data from the “Sentinel” series of multi-sensors as input and employs a covariance-guided feature matching method for spatial–spectral information fusion to mitigate the issues. Enhancing the spatial details of the S3 multi-band spectral channels preserves more spectral features of islands and adjacent sea areas. This is mainly reflected in considering the spectral residual scale information generated by the covariance between the detail image $I_{PL}$ and ${\tilde{MS}}_{k}$. This enables more accurate and comprehensive monitoring of ocean color.

The rest of the paper is structured as follows. In Section 3, we briefly introduce the pansharpening framework based on the injective regression scheme and the HPM injection scheme. Additionally, we present a proposed scheme on mixed-scale mutual information regression for the pansharpening of South China Sea islands from multi-source images. In Section 4, we present the experimental results, including quantitative and visual comparisons between the proposed method and some popular methods. Finally, in Section 5, we conclude the paper.

## 3. Methodology

In this section, we will review the discussion on the injection gain-based pansharpening framework and detail-scale regression, followed by an introduction to the proposed pansharpening method for island waters, MTF-GLP-HPM-HSMI.

### 3.1. Injection Gain and Detail Scale Regression

Let *P* and $MS_{k}$, with *k* ranging from 1 to *K*, denote the original PAN and MS images, respectively. Here, *k* signifies the spectral band index. The pansharpening injection scheme is formulated as follows:

(1) $${\hat{MS}}_{k}={\tilde{MS}}_{k}+g_{k}D={\tilde{MS}}_{k}+g_{k}\left(P-P_{L}\right),k=1,\ldots ,K.$$

where ${\hat{MS}}_{k}$ and ${\tilde{MS}}_{k}$ denote the fused MS images and the LRMS images (obtained by upsampling MS to the PAN scale using a 23-tap interpolation), respectively. $g_{k}$ represents the injection coefficients for the *k* th band. *D* is the detail image, and $P_{L}$ denotes the equivalent low-pass filtering (LPF) of *P* by generalized Laplacian pyramid (GLP) with MTF-matched filter.

Next, let us focus on the injection gains and use them to construct the detail-scale regression. The residual between REF and ${\hat{MS}}_{k}$ can be expressed as follows:

(2) $$REF_{k}-{\hat{MS}}_{k}=\left(REF_{k}-g_{k}P\right)-\left({\tilde{MS}}_{k}-g_{k}P_{L}\right).$$

That is, according to Wald’s protocol, the MS image generated by fusion should very closely approximate the ground-truth HRMS image, referred to as the reference (REF). To achieve this goal, an injection gain strategy is typically employed (injecting high-frequency details from the panchromatic image into the upsampled low-resolution multispectral image). Therefore, the two parentheses in Equation (2) represent the residuals of the MS and PAN images at the HR and LR scales, respectively. That is, the fusion result should align with the spectral characteristics of the original low-resolution multispectral (LRMS) image, and the spatial resolution of the fusion result should also be close to that of the high-resolution reference image (REF).

If both residuals are considered as the total loss of the estimate (i.e., independently, only one class is considered) [41], the respective optimal solutions are

(3) $$g_{k}^{\mathrm{LR}}=\arg\min_{g_{k}}{∥{\tilde{MS}}_{k}-g_{k}P_{L}∥}_{F}^{2}=\frac{\mathrm{cov}\left({\tilde{MS}}_{k},P_{L}\right)}{\mathrm{cov}\left(P_{L},P_{L}\right)},$$

(4) $${\hat{g}}_{k}^{\mathrm{HR}}=\arg\min_{g_{k}}{∥REF_{k}-g_{k}P∥}_{F}^{2}\approx \frac{\mathrm{cov}\left({\hat{MS}}_{k},P\right)}{\mathrm{cov}\left(P,P\right)},$$

where $\mathrm{cov}\left(A,B\right)$ denotes the covariance of the matrix, and the optimal gain is obtained by dividing the covariance values $g_{k}$, and ${∥\cdot ∥}_{F}$ denotes the Frobenius norm of a matrix. Therefore, the optimal injection gain at the LR scale can be interpreted as the scalar projection of ${\tilde{MS}}_{k}$ onto $P_{L}$. At the HR scale, however, ground truth is unavailable in practice, and the fused image approximates the reference image in spatial resolution. By replacing $REF_{k}$ with ${\hat{MS}}_{k}$, an approximate gain at the HR scale can be derived. At the same time, it should be noted that optimizations Equations (3) and (4) are band-dependent. This means that each band has its own optimal gain. This is because the spectral responses and noise levels vary across different bands, requiring independent calculations. To streamline the discussion, the specific indices of spectral bands will not be detailed.

By substituting the approximate gain Equation (4) into Equation (1), a new fused image can be obtained. By repeating the aforementioned steps, we obtain an iterative solution for ${\hat{g}}_{k}$.

(5) $${\hat{g}}_{k}=\frac{\mathrm{cov}\left({\tilde{MS}}_{k},P\right)}{\mathrm{cov}\left(P_{L},P\right)}.$$

In essence, however, these above estimates are considered as suboptimal solutions. According to [41], the best estimates are expressed in terms of detail images. Therefore, information at both scales, LR and HR, should be involved in the estimation, and the optimal solution of Equation (2) can be described as

(6) $$g_{k}=\frac{\mathrm{cov}\left({\tilde{MS}}_{k},D\right)}{\mathrm{cov}\left(P_{L},D\right)}.$$

In this work, we have designed an MRA model based on a detail scale regression. Based on the HS model [41], the optimality assumption for detail estimation was used, supplemented with spectral matching; to replace the detail image *D* with detail images that have undergone full-scale regression $I_{PL}$, rewrite Equation (6)

(7) $$g_{k}=\frac{\mathrm{cov}\left({\tilde{MS}}_{k},I_{PL}\right)}{\mathrm{cov}\left(P_{L},I_{PL}\right)},$$

where

(8) $$I_{PL}=\frac{{\tilde{MS}}_{k}}{P_{L}}D_{L}^{MS}.$$

Have undergone full-scale regression $I_{PL}$ here aims to eliminate radiometric differences between multispectral (MS) and panchromatic (PAN) images, normalizing the pixel values of the multispectral bands to the radiometric range of the panchromatic image, thereby suppressing spectral distortions in the fused results.

### 3.2. HPM Injection Scheme

In terms of injection gain, we choose an HPM injection scheme [32,42] that is different from the HS model. Therefore, Equation (1) can be rewritten as follows:

(9) $${\hat{MS}}_{k}={\tilde{MS}}_{k}+\frac{{\tilde{MS}}_{k}}{P_{L}}\left(P-P_{L}\right)={\tilde{MS}}_{k}\frac{P}{P_{L}}.$$

Next, we add detail scale regression to the HPM injection method by setting the digital MS or PAN image $D_{s}^{\mathrm{XR}}$ (either HR or LR) captured by the sensor to be defined as the convolution of the spatial response of the sensor with the total energy captured $S_{s}^{\mathrm{XR}}\left(x,y\right)$ by the sensor *s* in its spectral band $\omega_{s}\left(x,y\right)$:

(10) $$\begin{array}{cc} D_{s}^{\mathrm{XR}}\left(x,y\right) & =\epsilon_{s}+k_{s}\int_{-\infty }^{+\infty }\int_{-\infty }^{+\infty }S_{s}\left(x-\alpha ,y-\beta \right)\\ \; & \;\cdot \left(\int_{-\infty }^{+\infty }L\left(\alpha ,\beta ,\lambda \right)R_{s}\left(\lambda \right)d\lambda \right)d\alpha d\beta \\ \; & =\epsilon_{s}+k_{s}S_{s}^{\mathrm{XR}}\left(x,y\right)*\omega_{s}\left(x,y\right), \end{array}$$

where $\epsilon_{s}$ and $k_{s}$ are additive (or offset) and multiplicative (or gain) constants, respectively; $S_{s}$ is the spatial response of the sensor *s*, $\omega_{s}$ is the integral of the sensor’s radiated frequency weighted by the relative spectral response, and $\epsilon_{s}$ is usually negligible, so it will be disregarded in the following.

Based on Equations (9) and (10), ${\tilde{MS}}_{k}$, $P_{L}$, and *P* are now expressed as follows:

(11) $$\begin{array}{cc} {\tilde{MS}}_{k}=\; & k_{ms}S_{ms}^{\mathrm{LR}}*\omega_{ms}, \end{array}$$

(12) $$\begin{array}{cc} P_{L}=\; & k_{p}S_{PL}^{\mathrm{LR}}*\omega_{p}, \end{array}$$

(13) $$\begin{array}{cc} P=\; & k_{p}S_{P}^{\mathrm{HR}}*\omega_{p}, \end{array}$$

where, $S_{MS}^{\mathrm{LR}}$, $S_{PL}^{\mathrm{LR}}$, and $S_{P}^{\mathrm{HR}}$ denote the spatial responses of the LR-MS image, the LR-PAN image, and the HR-PAN image, respectively; $\omega_{ms}$ and $\omega_{p}$ denote the total energy of the corresponding sensor over its spectrum, respectively.

In this way, we obtain the HR MS image ${\hat{MS}}_{k}$ using the spatial response of HR-PAN $S_{P}^{\mathrm{HR}}$ and the total energy $\omega_{ms}$:

(14) $${\hat{MS}}_{k}=k_{ms}S_{P}^{\mathrm{HR}}*\omega_{ms}.$$

By substituting Equations (11)–(13) into Equation (9) for the HPM injection scheme, another form of ${\hat{MS}}_{k}$ is obtained:

(15) $${\hat{MS}}_{k}=\left(k_{ms}S_{ms}^{\mathrm{LR}}*\omega_{ms}\right)\frac{k_{p}S_{P}^{\mathrm{HR}}*\omega_{p}}{k_{p}S_{PL}^{\mathrm{LR}}*\omega_{p}}.$$

It can be observed that Equations (14) and (15) differ in variables other than ${\hat{MS}}_{k}$ and can be transformed into each other. Therefore, we can use tilde to distinguish between actual variables and estimated variables. Thus, the modified equations are as follows:

(16) $${\hat{MS}}_{k}={\tilde{MS}}_{k}\frac{\tilde{P}}{\tilde{P_{L}}}=\left(k_{ms}S_{ms}^{\mathrm{LR}}*\omega_{ms}\right)\frac{k_{p}{\tilde{S}}_{P}^{\mathrm{HR}}*{\tilde{\omega }}_{p}}{k_{p}{\tilde{S}}_{PL}^{\mathrm{LR}}*{\tilde{\omega }}_{p}}.$$

As a result, the following equation is obtained.

(17) $${\tilde{S}}_{P}^{\mathrm{HR}}=S_{P}^{\mathrm{HR}}.$$

(18) $${\tilde{S}}_{PL}^{\mathrm{LR}}=S_{ms}^{\mathrm{LR}}.$$

This is because the virtual sensor that ultimately generates the HRMS image should have the same spatial response as the existing PAN sensor, while the construction of the $P_{L}$ is supported by a filter that matches the spatial response of the MS sensor.

Based on [36], the third equation is defined as

(19) $$k_{p}{\tilde{\omega }}_{p}=k_{ms}\omega_{ms}.$$

Thus, considering Equations (11) and (12), we obtain Equation (20)

(20) $$\begin{array}{cc} \tilde{P_{L}}= & k_{p}{\tilde{S}}_{PL}^{\mathrm{LR}}*{\tilde{\omega }}_{p}\\ = & k_{ms}S_{ms}^{\mathrm{LR}}*\omega_{ms}={\tilde{MS}}_{k}. \end{array}$$

Similarly, we obtain

(21) $$\begin{array}{cc} \tilde{P}= & k_{p}{\tilde{S}}_{P}^{\mathrm{HR}}*{\tilde{\omega }}_{p}\\ = & k_{ms}S_{P}^{\mathrm{HR}}*\omega_{ms}={\hat{MS}}_{k}. \end{array}$$

Combining Equations (20) and (21), a linear affine function is proposed to solve ${\hat{MS}}_{k}^{\mathrm{XR}}$ as follows:

(22) $${\tilde{P}}^{\mathrm{XR}}=mP^{\mathrm{XR}}+n={\hat{MS}}_{k}^{\mathrm{XR}}.$$

**Figure 2:**
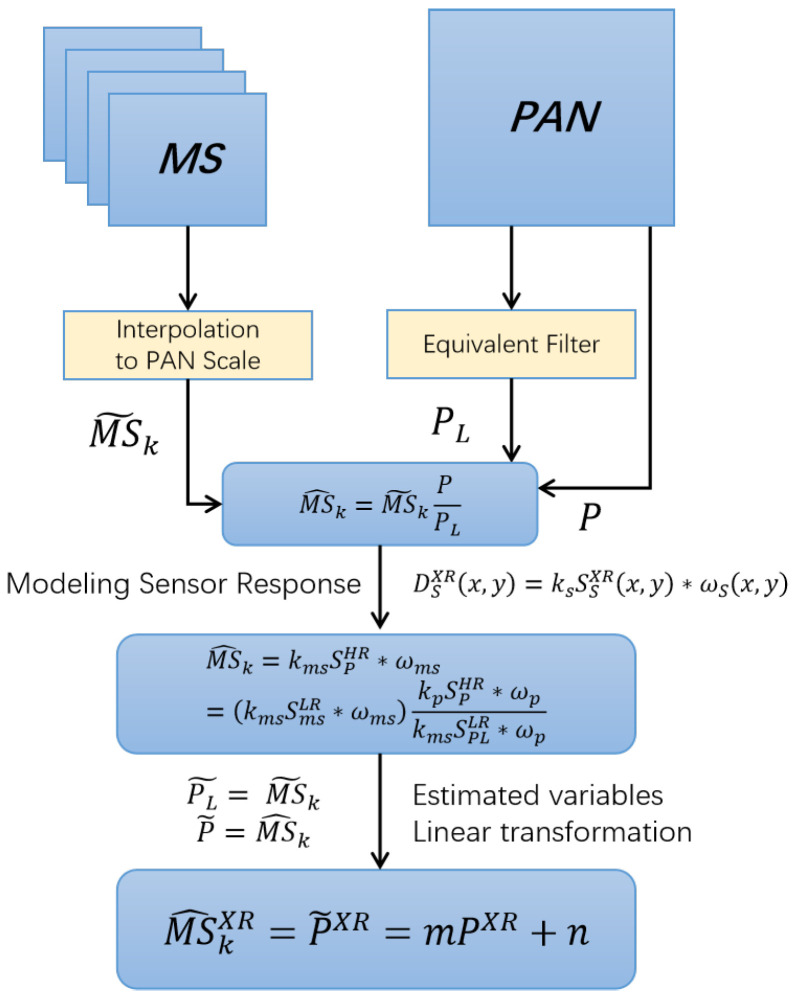
Schematic diagram of HPM injection scheme.

Figure 2 shows a schematic diagram related to the HPM injection scheme.

### 3.3. Mixed-Scale Regression for Detailed Images

Thus, according to Equation (22), the problem is transformed into finding the coefficients *m* and *n* to obtain ${\hat{MS}}_{k}^{\mathrm{XR}}$. In this paper, spectral matching between LR PAN and MS data is established through linear regression, where the coefficients *m* and *n* of the linear model are estimated using the least squares method [36].

(23) $$\begin{array}{cc} m=\; & \frac{\mathrm{cov}\left({\tilde{MS}}_{k},I_{PL}\right)}{\mathrm{cov}\left(P_{L},I_{PL}\right)}, \end{array}$$

(24) $$\begin{array}{cc} n=\; & E\left({\tilde{MS}}_{k}\right)-\frac{\mathrm{cov}\left({\tilde{MS}}_{k},I_{PL}\right)}{\mathrm{cov}\left(P_{L},I_{PL}\right)}E\left(P\right), \end{array}$$

where E(*X*) represents the mean of image *X*.

Thus, we can rewrite Equation (16):

(25) $$\begin{array}{cc} {\hat{MS}}_{k}=\; & {\tilde{MS}}_{k}\frac{\tilde{P}}{\tilde{P_{L}}}\\ =\; & {\tilde{MS}}_{k}\frac{P+C_{b}}{P_{L}+C_{b}}, \end{array}$$

where

(26) $$C_{b}=E\left({\tilde{MS}}_{k}\right)/g_{k}-E\left(P\right).$$

To address the single-scale deficiency of scale regression-based methods [35], we enhance these methods with detailed image information and construct a hybrid-scale regression. This approach not only considers the spectral residual scale information from the covariance between the detail image $I_{PL}$, which has undergone full-scale regression, and ${\tilde{MS}}_{k}$, but also considers the spatial residual scale information from the covariance between $I_{PL}$ and *P*. The covariance-guided matching of remote sensing data features originating from different sensors is so realized that “HSMI” can be obtained:

(27) $$g_{k}^{i}=MI\frac{\mathrm{cov}\left({\tilde{MS}}_{k}^{i},I_{PL}\right)}{\mathrm{cov}\left(I_{PL},P_{L}\right)}+\left(1-MI\right)\frac{\mathrm{cov}\left(P,I_{PL}\right)}{\mathrm{cov}\left(I_{PL},P_{L}\right)},$$

where MI is based on the computationally generated mutual information of the detail image $I_{PL}$ with $P_{L}$, as follows:

(28) $$MI\left(I_{PL};P_{L}\right)=\sum_{x\in I_{PL}}\sum_{y\in P_{L}}p\left(x,y\right)\cdot \log_{2}\left(\frac{p(x,y)}{p(x)\cdot p(y)}\right),$$

Here, p(x), p(y), and p(x,y) denote the marginal probability distribution function and the joint probability distribution function for $I_{PL}$ and $P_{L}$, respectively. Such control parameters are more expressive of the intrinsic representation between the detailed image and the degraded PAN image to balance the fusion representation at the mixed scale.

In addition, the input PAN and MS images do not correspond to each other due to the inherent bias of multi-source sensor platforms. The spectral matching of $I_{PL}$ and ${\tilde{MS}}_{k}$ is performed before injecting the gain estimation so that they have almost the same statistical characteristics:

(29) $$I_{PL}=\frac{{\tilde{MS}}_{k}}{P_{L}}D_{L}^{MS}=\frac{{\tilde{MS}}_{k}}{P_{L}}\left(\left(a_{k}P+b_{k}\right)-\left(a_{k}P_{L}+b_{k}\right)\right),$$

where

(30) $$a_{k}=\frac{\mathrm{std}({\tilde{MS}}_{k})}{\mathrm{std}(P_{L})},\;b_{k}=\mu_{{\tilde{MS}}_{k}}-a_{k}\mu_{P_{L}},$$

Meanwhile, μ and std denote the mean and standard deviation between the matched images, respectively.

Figure 1 shows the flowchart of the MTF-GLP-HPM-HSMI pansharpening method. After each iteration, the injection coefficient $g_{k}$ is obtained, which is substituted into Equation (25) to obtain the pansharpening result. When the iterative process is stopped, the final pansharpening result is obtained. The process is described in Algorithm 1.
**Algorithm 1** MTF-GLP-HPM-HSMI  **Input**: An original MS image and an original PAN image;  (1) Interpolate the MS image to the size of the PAN image;  (2) Obtain $P_{L}$ using the PAN image using the MTF-GLP;  **for** each band k∈{1,…,K} **do**
   1. Calculate the gain coefficients $g_{k}$ using Equation (27);   2. Spectral matching was performed using Equation (29) prior to estimation and detailed images were generated $I_{PL}$;   3. By using Equation (28) to calculate the mutual information between $P_{L}$ and $I_{PL}$, the mixed weighting coefficient MI is derived;   **for** each iteration i∈{0,…,N−1} **do**
  1. Calculate injection coefficients $g_{k}^{i}$ as:     $g_{k}^{i}\leftarrow MI\frac{\mathrm{cov}\left({\tilde{MS}}_{k}^{i},I_{PL}\right)}{\mathrm{cov}\left(I_{PL},P_{L}\right)}+\left(1-MI\right)\frac{\mathrm{cov}\left(P,I_{PL}\right)}{\mathrm{cov}\left(I_{PL},P_{L}\right)}$  2. Use $g_{k}^{i}$ to fuse the MS and PAN as follows:     ${\hat{MS}}_{k}^{i}\leftarrow {\tilde{MS}}_{k}\frac{P+C_{b}}{P_{L}+C_{b}}$     $C_{b}=E\left({\tilde{MS}}_{k}\right)/g_{k}^{i}-E\left(P\right)$   **end for**   Stop the iterative process and output ${\hat{MS}}_{k}$;  **end for**  **return** $\hat{MS}$

## 4. Experimental Results

### 4.1. Datasets and Preprocessing

**Figure 3:**
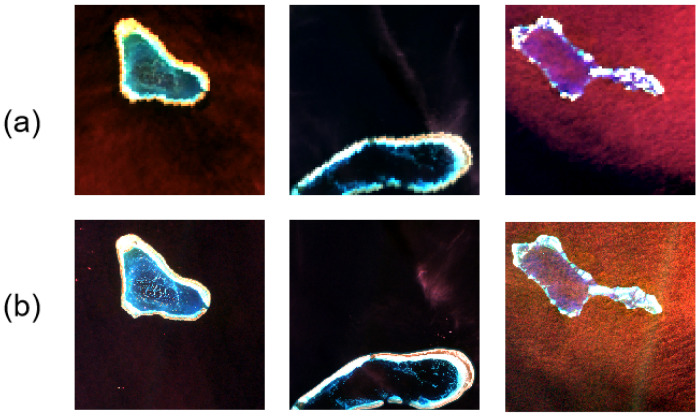
Visualization of the considered datasets made of coupled S3 OLCI (**a**) and S2 MSI scenes (**b**).

This work consists of three distinct datasets of paired S2-MSI and S3-OLCI products covering several key island regions in the South China Sea, including Huangyan Island, Sabina Shoal, and Discovery Reef. Table 1 summarizes the selected scenes and their acquisition dates and locations. The datasets chosen for construction were all cloud-free products downloaded from the Copernicus Open Access Centre and preprocessed using the Sentinel Application Platform (SNAP). For S2, the Level 2B MSI images were spatially resampled to 20 m using the Sen2Cor processor to generate homogeneous data cubes for HR panchromatic inputs. Atmospheric corrections were performed for the S3-OLCI images used as multispectral inputs. Finally, the OLCI images were reprojected onto the corresponding S2 plots to capture the overlap region between the two sensors of the construct (see Figure 3).

In this section, we define the input and output images of the considered multi-source inter-sensor pansharpening scheme. They are input LRMS ($I_{MS}$) and input HR panchromatic ($I_{PAN}$). In this study, we only consider MSI and OLCI bands with similar wavelengths, i.e., the red (R), green (G), blue (B), and near-infrared (IR) bands at wavelengths of 665, 560, 490, and 865 nm. Therefore, $I_{MS}$ is defined as Oa04, Oa06, Oa08, Oa17. In addition, a 21-band extension of $I_{MS}$ was performed to verify the spectral retention adaptation of the proposed method in multispectral fusion. In addition, $I_{PAN}$ is generated by calculating the corresponding bands of MSI by band averaging and adjusting the spatial size to R × OLCI (R = 4 in this paper).

### 4.2. Benchmarks and Assessment

To validate the advantages of the proposed MTF-GLP-HPM-HSMI method, we selected the popular pansharpening methods from different categories for benchmarking, with a particular focus on detailed benchmarking of the MTF-GLP class pansharpening methods within MRA, including C-GSA [43], BDSD-PC [44], AWLP [19], MF [20], MTF-GLP [21], MTF-GLP-HPM [42], MTF-GLP-HPM-H [45], MTF-GLP-HPM-R [36], MTF-GLP-CBD [33], MTF-GLP-Reg-FS [34], RR [46], TV [47], FE-HPM [48], and PWMBF [49].

To provide a comprehensive analysis of the experimental results presented in this paper, both down-resolution experiments (RR) and full-resolution experiments (FR) were conducted on all three South China Sea island datasets. In the down-resolution experiments, the original MS is used as the ideal fusion result according to the synthetic properties of the Wald protocol. The down-sampled and then up-sampled MS and down-sampled PAN are used as the inputs, and the results obtained from the experiments are evaluated in comparison with the original MS to obtain the index results. The primary advantage is the objective assessment of quality distortion measurements. However, there is also a clear disadvantage, namely the assumption of scale invariance. To address this issue, we used full-resolution experiments to aid in the validation, where RR experiments were selected as evaluation metrics for $Q2^{n}$ (Universal Image Quality Index), ERGAS (relative dimensionless global error index), SAM (spectral angle mapper), and PSNR (Peak Signal-to-Noise Ratio). The FR evaluation was used as the Hybrid Quality Without Reference (HQNR) index. It consists of the product of two independent values $D_{\lambda }^{k}$ and $D_{S}$ that quantify the spectral and spatial distortions:

(31) $$\begin{array}{c} \;\left\{\begin{array}{cc} \; & D_{\lambda }^{k}=1-Q2^{n}\left({\hat{MS}}_{d},MS\right)\\ \; & D_{s}=\frac{1}{K}\sum_{k=1}^{K}\left|Q\left({\hat{MS}}_{k},P\right)-Q\left({\tilde{MS}}_{k},P_{L}\right)\right|\\ \; & HQNR=\left(1-D_{\lambda }^{k}\right)\left(1-D_{S}\right) \end{array}\right.\; \end{array}$$

where ${\hat{MS}}_{d}$ represents the degraded version of the fused outcome, and *Q* denotes the Universal Image Quality Index (UIQI). The HQNR metric ranges between 0 and 1, with higher values indicating superior pansharpening quality. This index effectively combines spectral and spatial information to assess the quality of pansharpening images without requiring a reference image. In the quantitative assessment, the optimal values for the selected metrics are as follows: $Q2^{n}$ (1), ERGAS (0), SAM (0), PSNR (+∞), $D_{\lambda }$ (0), $D_{s}$ (1), and HQNR (1); In addition, calculating time (CT) is also included in the evaluation to demonstrate computational efficiency.

### 4.3. Quantitative Comparison Results

Table 2, Table 3 and Table 4 summarize the quantitative results for the three pairs of coupled data (i.e., HY, XB, and HG). In this paper, “HSMI” stands for the method MTF-GLP-HPM-HSMI. In addition, the two best results for each indicator are highlighted in bold.

Comprehensive evaluations under reduced-resolution (RR) and full-resolution (FR) protocols indicate that, compared to other regression-based multi-resolution analysis (MRA) methods (e.g., MTF-GLP-CBD, MTF-GLP-HPM, and MTF-GLP-Reg-FS), the HSMI method performs favorably in terms of spectral fidelity. To further examine the fusion performance, we analyzed the metrics across three aspects based on their characteristics: (1) spectral distortion, (2) spatial distortion, and (3) spatial–spectral coupling. The first group of metrics, including SAM and $D_{\lambda }^{k}$, quantifies spectral errors. On the HY, XB, and HG datasets, HSMI achieves the lowest SAM values of 0.3332, 0.5600, and 0.3708, respectively; under FR evaluation, its $D_{\lambda }^{k}$ values are 0.2169, 0.2126, and 0.1830, suggesting effective preservation of spectral information. The second group, comprising $D_{S}$ and ERGAS, assesses spatial detail quality and distortion. HSMI’s fused images exhibit relatively clear island edges and improved detail resolution; for instance, on the HY dataset, its $D_{S}$ value is 0.0341, slightly higher than BDSD-PC (0.0053) or TV (0.0122), yet its overall spatial performance remains competitive. The third group, including $Q2^{n}$ and HQNR, evaluates spatial–spectral coupling. HSMI records $Q2^{n}$ values of 0.7707, 0.8776, and 0.8945, and HQNR values of 0.7563, 0.7697, and 0.8071 across the HY, XB, and HG datasets, respectively, ranking among the top and demonstrating stable performance compared to methods like BDSD-PC, MTF-GLP-Reg-FS, and AWLP. Overall, these results suggest that HSMI achieves a suitable fusion of spatial and spectral resolution in island sea areas environments, effectively balancing spectral fidelity and spatial detail enhancement.

### 4.4. Visual and Qualitative Comparisons

**Figure 4:**
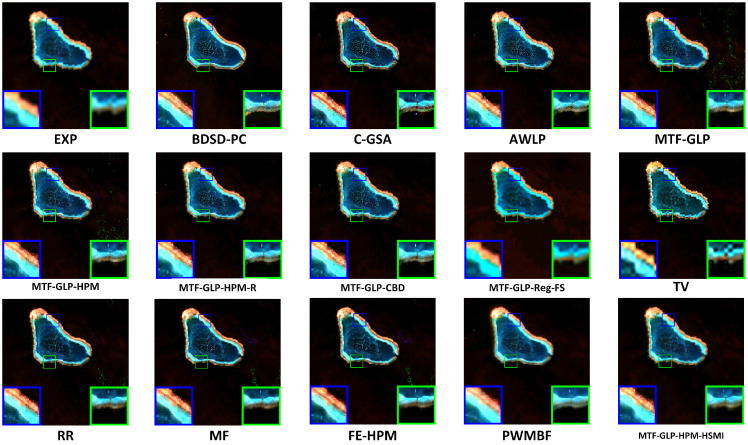
Visual presentation of the HY experiment (true color).

**Figure 5:**
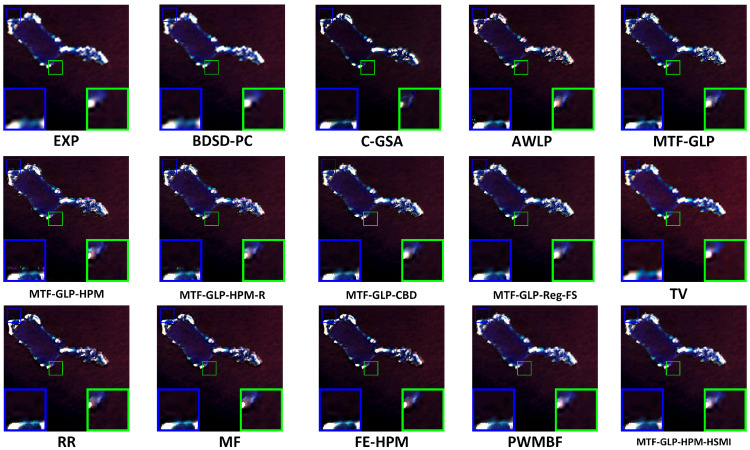
Visual presentation of the XB experiment (true color).

**Figure 6:**
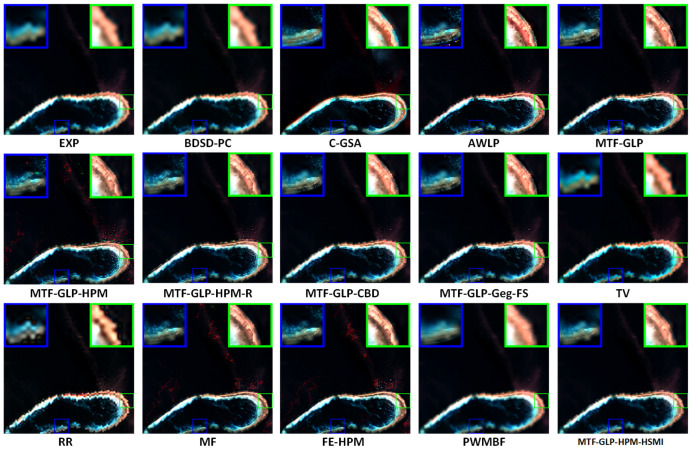
Visual presentation of the HG experiment (true color).

Figure 4, Figure 5 and Figure 6, respectively, present the visual results of experiments conducted on three coupled Sentinel-2 and Sentinel-3 datasets (HY, XB, and HG). The fusion results are displayed in true color, specifically the Oa04, Oa06, and Oa08 bands of Sentinel-3. Compared with variational optimization (VO)-based methods (such as TV and RR), component substitution (CS)-based methods (such as BDSD-PC and C-GSA) generally preserve spatial details more effectively. However, CS methods exhibit limitations in spectrally sensitive scenarios, while VO methods often suffer from significant spectral distortion and spatial detail loss, partly due to their reliance on manual parameter adjustment, which reduces flexibility and increases computational complexity. MTF-GLP and other regression-based methods also show a certain degree of spectral distortion, as evidenced by the presence of obvious noise in the fusion results of MTF-GLP and its derivatives (such as MTF-GLP-HPM and MTF-GLP-Reg-FS), especially in spectrally sensitive areas, which further exacerbates spectral distortion.

Meanwhile, the magnified regions highlighted by the blue and green boxes indicate that the proposed HSMI method achieves a better balance between spatial detail clarity and spectral fidelity. Methods such as C-GSA, MTF-GLP, and regression-based methods (such as MTF-GLP-CBD and MTF-GLP-Reg-FS) show obvious spectral distortion in island environments, occasionally accompanied by noise artifacts, which may be due to their insufficient handling of the spatial heterogeneity of multi-source data. For instance, C-GSA shows excessive spectral smoothing in the green magnified box of the HG dataset, causing the island edge areas to appear overly green. Other VO-based methods and hybrid methods (such as FE-HPM, PWMBF, etc.) exhibit significant fusion noise on the HY and HG datasets. In addition, the visualization output results of BDSD-PC are inconsistent with the quantitative indicators. Although it performs well in spatial indicators (such as $D_{S}$: 0.0053 for the HY dataset), its fused images have halo effects and incorrect contour lines, which leads to the loss of island boundary features. This is in line with the lower $Q2^{n}$ value (HY: 0.6926). In contrast, the proposed HSMI in this paper shows clear textures and accurate color restoration in both island edges and lagoon areas, indicating that HSMI can effectively capture the complex features of island and sea scenes. This may be due to its adaptive regression framework, which can better adapt to the spectral and spatial characteristics of Sentinel-2 and Sentinel-3 data.

### 4.5. Extended Comparison

**Figure 7:**
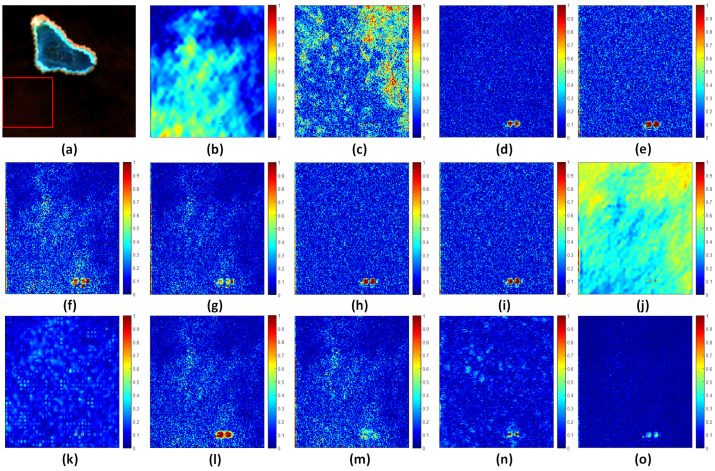
Subareas of full-band SAM average error for HY experiment. From left to right: (**a**) Selected subregions. (**b**) BDSD-PC. (**c**) C-GSA. (**d**) AWLP. (**e**) MTF-GLP. (**f**) MTF-GLP-HPM. (**g**) MTF-GLP-HPM-H. (**h**) MTF-GLP-HPM-R. (**i**) MTF-GLP-CBD. (**j**) MTF-GLP-Reg-FS. (**k**) TV. (**l**) RR. (**m**) MF. (**n**) FE-HPM. (**o**) MTF-GLP-HPM-HSMI.

**Figure 8:**
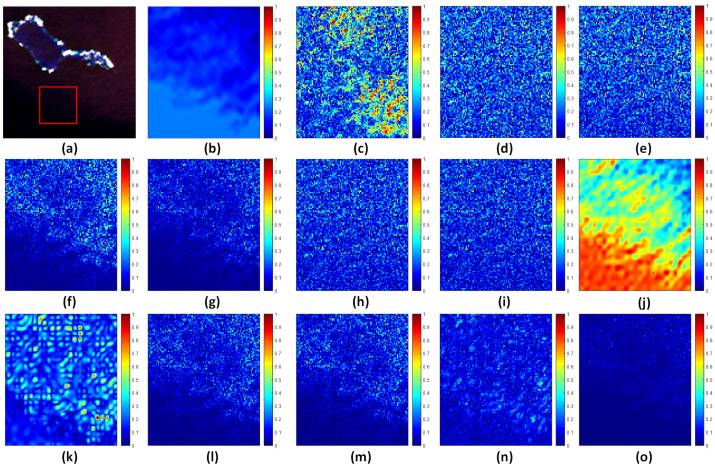
Subareas of full-band SAM average error for XB experiment. From left to right: (**a**) Selected subregions. (**b**) BDSD-PC. (**c**) C-GSA. (**d**) AWLP. (**e**) MTF-GLP. (**f**) MTF-GLP-HPM. (**g**) MTF-GLP-HPM-H. (**h**) MTF-GLP-HPM-R. (**i**) MTF-GLP-CBD. (**j**) MTF-GLP-Reg-FS. (**k**) TV. (**l**) RR. (**m**) MF. (**n**) FE-HPM. (**o**) MTF-GLP-HPM-HSMI.

**Figure 9:**
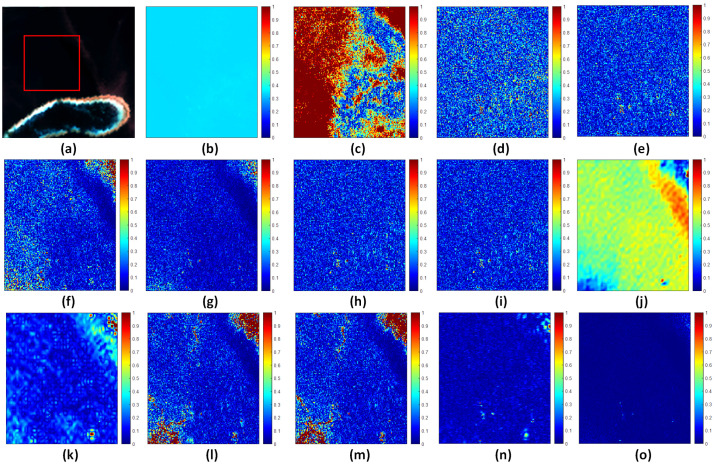
Subareas of full-band SAM average error for HG experiment. From left to right: (**a**) Selected subregions. (**b**) BDSD-PC. (**c**) C-GSA. (**d**) AWLP. (**e**) MTF-GLP. (**f**) MTF-GLP-HPM. (**g**) MTF-GLP-HPM-H. (**h**) MTF-GLP-HPM-R. (**i**) MTF-GLP-CBD. (**j**) MTF-GLP-Reg-FS. (**k**) TV. (**l**) RR. (**m**) MF. (**n**) FE-HPM. (**o**) MTF-GLP-HPM-HSMI.

To more clearly illustrate the differences in pansharpening images with respect to spectral fidelity and spatial detail enhancement and to further validate the superiority of the proposed HSMI method in mitigating spectral distortions in marine regions, we generated SAM (spectral angle mapper) error maps for subregions of the HY, HB, and HG datasets, as shown in Figure 7, Figure 8, and Figure 9, respectively. The results demonstrate that, compared to other methods, the fused images produced by the HSMI method (labeled as (o) in Figure 7, Figure 8 and Figure 9) exhibit the closest resemblance to the reference images derived from the original multispectral inputs, with significantly lower SAM errors. This strongly confirms the HSMI method’s capability to effectively balance spatial detail enhancement and spectral information preservation in complex marine environments.

**Figure 10:**
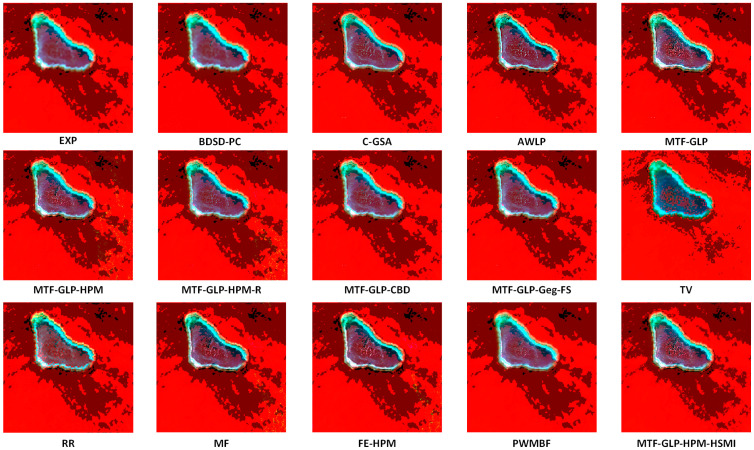
The results of the 4-band fusion of the HY dataset are shown in standard false color (NIR–red–green).

To systematically evaluate the performance of pansharpened images in terms of multi-band spectral fidelity and spatial detail enhancement, we first conducted a 4-band pansharpening experiment on the HY dataset, presenting the fused results in false-color format (Figure 10). The experimental results demonstrate that the proposed HSMI method achieves an excellent balance between spatial detail enhancement in island regions and spectral information preservation in surrounding marine areas, fully showcasing its superior capability in synergistic optimization of spectral and spatial features.

**Figure 11:**
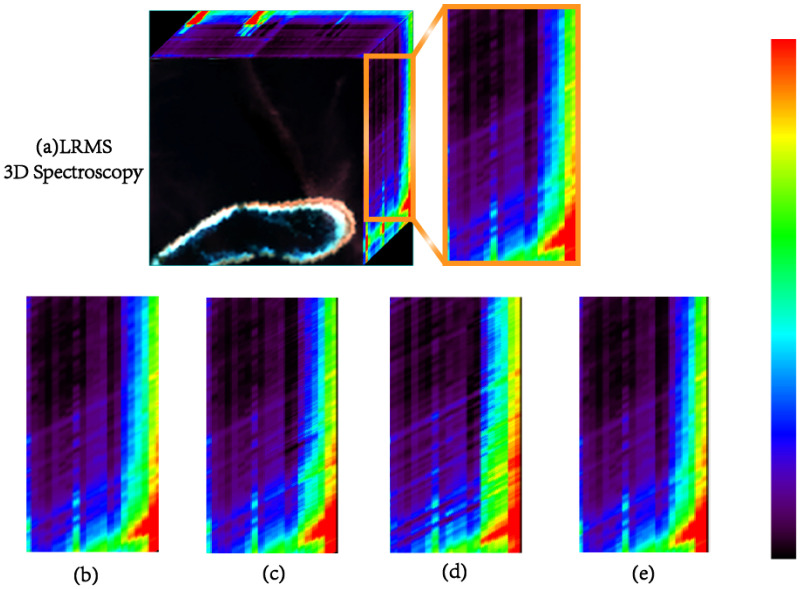
Spectral errors of 21-band experiments on the HG dataset. From left to right: (**a**) Selected subregions. (**b**) BDSD-PC. (**c**) MTF-GLP-Reg-FS. (**d**) RR. (**e**) MTF-GLP-HPM-HSMI.

To further validate the adaptability and robustness of the HSMI method in more complex multi-band scenarios, we performed an extended 21-band pansharpening experiment on the HG dataset, where the low-resolution multispectral image LRMS($I_{MS}$) was replaced with a full-band S3 image. The visual results and quantitative evaluations are presented in Figure 11 and Table 5, respectively, showing consistency with the 4-band experiment. Notably, even under challenging conditions where the original LRMS and PAN images in the HG dataset were affected by cloud and fog interference, the HSMI method maintained optimal spectral-related metrics. Although its spatial performance was slightly below the ideal level, no coupling effect between spectral and spatial distortions was observed. Comprehensive evaluation indicates that the HSMI method exhibits robust performance in multi-band pansharpening tasks, effectively addressing the demands for spectral fidelity and spatial enhancement in complex environments.

## 5. Conclusions

This paper introduces a multi-source pansharpening method based on mutual information hybrid-scale regression (HSMI), aimed at addressing the challenges of spectral fidelity and spatial detail optimization in multi-source remote sensing data fusion for island sea areas. Limited by the low spatial resolution of Sentinel-3 OLCI, systematic biases between sensor platforms, and spectral distortions caused by dynamic marine water changes due to inconsistent imaging times, conventional methods often yield suboptimal spectral preservation in marine regions when integrating Sentinel-2 spatial information with Sentinel-3 spectral information. To tackle these issues, we propose a hybrid scale regression framework that employs mutual information to quantify spatial–spectral correlations across multi-source data, computing weight parameters to integrate hybrid scale information and thereby producing high-precision pansharpened outputs.

To validate the performance of the proposed HSMI method, we utilized a South China Sea island dataset derived from coupled Sentinel-2 and Sentinel-3 data, conducting a systematic evaluation of reduced-resolution (ERGAS, SAM, PSNR, and $Q2^{n}$ metrics) and full-resolution ($D_{\lambda }^{k}$, $D_{S}$, and HQNR metrics) performance. The experimental results demonstrate that, compared to established mainstream pansharpening methods, the HSMI method (denoted as MTF-GLP-HPM-HSMI) enhances spatial details and edge clarity in island regions while markedly improving spectral fidelity in surrounding marine areas, showcasing exceptional spatial–spectral synergistic optimization. Although the current achievements have shown initial promise, there is still room for further optimization. In future work, we intend to explore advanced deep learning techniques, extend the approach to other multi-source sensor platforms, and apply it to water color inversion tasks in island sea areas, thereby enhancing its practical value in marine remote sensing applications.

## Figures and Tables

**Table 1:** Description of the constructed dataset.

Name	Scene	Sensing Dates		Tile (Ref. S2)
		S3	S2	
HY (Huangyan Island)	the Zhongsha Islands	21/03/2023	20/03/2023	50PNB
XB (Sabina Shoal)	the Nansha Islands	05/03/2024	05/03/2024	50PMR
HG (Discovery Reef)	the Xisha Islands	14/01/2023	14/01/2023	49QEU

**Table 2:** Quantitative evaluation results for the HY dataset.

Method	RR				FR			
	*Q*2^*n*^	*SAM* ↓	*ERGAS* ↓	*PSNR*	$D_{\lambda }^{k}↓$	*D_S_* ↓	*HQNR*	*CT*
GT/EXP	1.0000	0.0000	0.0000	—	0.1870	0.0010	0.8123	—
BDSD-PC	**0.6926**	7.1359	**2.6272**	**44.7982**	0.4471	**0.0053**	0.5499	0.26
C-GSA	0.2787	13.8578	16.5020	29.4042	0.6642	0.0270	0.3267	0.53
AWLP	0.5706	4.2464	16.9353	28.1994	**0.2420**	0.0318	**0.7339**	0.23
MF	0.4403	6.6867	98.0534	11.6006	0.3755	0.0354	0.6024	0.28
MTF-GLP	0.4987	5.5852	10.5662	33.0922	0.2948	0.0338	0.6814	0.10
MTF-GLP-HPM	0.3694	9.0922	532.5727	−3.2068	0.4252	0.0326	0.5561	0.11
MTF-GLP-HPM-R	0.4416	7.8198	308.4144	1.5485	0.4041	0.0233	0.5820	0.07
MTF-GLP-CBD	0.5594	5.3341	9.5631	33.7128	0.2883	0.0239	0.6947	0.09
MTF-GLP-Reg-FS	0.5618	5.2926	9.4667	33.8007	0.2848	0.0236	0.6983	0.09
TV	0.2828	10.1988	10.6424	35.0224	0.7937	**0.0122**	0.2038	5.65
RR	0.5578	5.6050	9.7046	33.8492	0.4556	0.0151	0.5362	4.42
FE-HPM	0.4229	6.9582	397.6030	1.4545	0.4123	0.0371	0.5659	0.42
PWMBF	0.5312	**4.1260**	**7.5496**	**37.3347**	0.4788	0.0390	0.5009	0.47
HSMI	**0.7707**	**0.3332**	7.8479	35.7845	**0.2169**	0.0341	**0.7563**	0.20

**Table 3:** Quantitative evaluation results for the XB dataset.

Method	RR				FR			
	*Q*2^*n*^	*SAM* ↓	*ERGAS* ↓	*PSNR*	$D_{\lambda }^{k}↓$	*D_S_* ↓	*HQNR*	*CT*
GT/EXP	1.0000	0.0000	0.0000	—	0.1989	0.0019	0.7995	—
BDSD-PC	**0.8167**	3.6684	**0.8800**	**59.8759**	0.2988	**0.0021**	0.6998	0.05
C-GSA	0.5357	7.8938	30.8173	28.8323	0.4937	0.0209	0.4958	0.26
AWLP	0.5749	4.8236	36.1730	26.5874	0.2610	0.0223	0.7225	0.15
MF	0.7115	**1.6490**	20.4871	32.4754	0.2684	0.0311	0.7088	0.04
MTF-GLP	0.6142	4.7505	17.6212	33.8376	0.2603	0.0295	0.7178	0.07
MTF-GLP-HPM	0.5821	2.6724	76.3874	21.6583	0.2744	0.0283	0.7051	0.08
MTF-GLP-HPM-R	0.7511	1.7318	146.2509	16.0651	0.2488	0.0204	0.7359	0.05
MTF-GLP-CBD	0.7261	3.7199	13.4657	36.0038	0.2467	0.0214	0.7371	0.05
MTF-GLP-Reg-FS	0.7367	3.5922	12.9381	36.3705	**0.2427**	0.0210	**0.7414**	0.07
TV	0.5230	10.4948	10.8895	**40.4000**	0.5157	0.0068	0.4810	2.62
RR	0.6264	6.5751	13.9354	35.9274	0.3473	**0.0066**	0.6484	3.89
FE-HPM	0.6878	1.7861	27.6523	29.8884	0.2935	0.0331	0.6830	0.20
PWMBF	0.6889	3.0461	**9.3035**	39.7592	0.3850	0.0288	0.5973	0.24
HSMI	**0.8776**	**0.5600**	12.0783	37.3864	**0.2126**	0.0226	**0.7697**	0.08

**Table 4:** Quantitative evaluation results for the HG dataset.

Method	RR				FR			
	*Q*2^*n*^	*SAM* ↓	*ERGAS* ↓	*PSNR*	$D_{\lambda }^{k}↓$	*D_S_* ↓	*HQNR*	*CT*
GT/EXP	1.0000	0.0000	0.0000	—	0.1742	0.0010	0.8250	—
BDSD-PC	0.6967	3.3546	**1.4289**	**51.5819**	0.4442	**0.0029**	0.5542	0.04
C-GSA	0.3733	13.1861	27.7954	25.5358	0.6680	0.0169	0.3263	0.20
AWLP	0.6249	4.6637	40.9876	22.0931	0.2800	0.0142	0.7097	0.12
MF	0.4875	4.8841	307.9827	12.0675	0.3513	0.0242	0.6331	0.04
MTF-GLP	0.6095	3.8790	13.1801	33.5947	0.2700	0.0222	0.7138	0.07
MTF-GLP-HPM	0.4186	5.8181	248.6191	9.3156	0.3600	0.0191	0.6278	0.07
MTF-GLP-HPM-R	0.7142	**1.6330**	50.3957	23.2851	**0.2232**	0.0199	**0.7614**	0.05
MTF-GLP-CBD	0.6731	3.2091	10.0218	34.8990	0.2509	0.0202	0.7340	0.05
MTF-GLP-Reg-FS	0.6764	3.1540	9.8490	35.0432	0.2494	0.0200	0.7355	0.05
TV	0.5180	8.1571	25.3791	34.6124	0.5676	**0.0063**	0.4296	2.31
RR	0.6732	5.8643	13.6862	33.4933	0.3812	0.0069	0.6145	3.64
FE-HPM	0.4445	5.2907	1468.0778	−0.5753	0.3953	0.0261	0.5890	0.19
PWMBF	**0.7728**	2.0174	**7.5237**	**38.5400**	0.3108	0.0197	0.6756	0.28
HSMI	**0.8945**	**0.3708**	15.4491	31.9907	**0.1830**	0.0122	**0.8071**	0.08

**Table 5:** Results of the 21-band extension experiment on the HG dataset.

Method	RR				FR			
	*Q*2^*n*^	*SAM* ↓	*ERGAS* ↓	*PSNR*	$D_{\lambda }^{k}↓$	*D_S_* ↓	*HQNR*	*CT*
GT/EXP	1.0000	0.0000	0.0000	—	0.1549	0.0013	0.8440	—
BDSD-PC	0.7318	2.9355	**1.0535**	**54.7527**	0.4124	**0.0025**	0.5862	0.57
MTF-GLP-Reg-FS	0.6846	2.4776	7.1824	**38.9821**	0.2542	0.0237	0.7281	0.35
RR	0.5214	9.7966	15.4098	34.6199	0.5658	0.0122	0.4289	17.78
HSMI	**0.8669**	**0.5118**	14.2184	37.6199	**0.1677**	0.0171	**0.8180**	0.63

## Data Availability

The data presented in this study are available on request from the corresponding author.

## References

[1] Tian Y., Duan M., Cui X., Zhao Q., Tian S., Lin Y., Wang W. (2023). Advancing application of satellite remote sensing technologies for linking atmospheric and built environment to health. Front. Public Health.

[2] Dube T., Shekede M.D., Massari C. (2022). Remote sensing for water resources and environmental management. Remote Sens..

[3] Ma F., Huo S., Yang F. (2021). Graph-based logarithmic low-rank tensor decomposition for the fusion of remotely sensed images. IEEE J. Sel. Top. Appl. Earth Obs. Remote Sens..

[4] Wu K., Chen T., Xu Y., Song D., Li H. (2021). A Novel Change Detection Approach Based on Spectral Unmixing from Stacked Multitemporal Remote Sensing Images with a Variability of Endmembers. Remote Sens..

[5] Chawla I., Karthikeyan L., Mishra A.K. (2020). A review of remote sensing applications for water security: Quantity, quality, and extremes. J. Hydrol..

[6] Yang H., Kong J., Hu H., Du Y., Gao M., Chen F. (2022). A review of remote sensing for water quality retrieval: Progress and challenges. Remote Sens..

[7] Concha J.A., Schott J.R. (2016). Retrieval of color producing agents in Case 2 waters using Landsat 8. Remote Sens. Environ..

[8] Soppa M.A., Silva B., Steinmetz F., Keith D., Scheffler D., Bohn N., Bracher A. (2021). Assessment of polymer atmospheric correction algorithm for hyperspectral remote sensing imagery over coastal waters. Sensors.

[9] Yu G., Zhong Y., Fu D., Chen F., Chen C. (2024). Remote sensing estimation of *δ*15NPN in the Zhanjiang Bay using Sentinel-3 OLCI data based on machine learning algorithm. Front. Mar. Sci..

[10] Liu Z., Han X.H. (2024). Hyperspectral image super resolution using deep internal and self-supervised learning. CAAI Trans. Intell. Technol..

[11] Wang S., Jiang X., Spyrakos E., Li J., McGlinchey C., Constantinescu A.M., Tyler A.N. (2024). Water color from Sentinel-2 MSI data for monitoring large rivers: Yangtze and Danube. Geo-Spat. Inf. Sci..

[12] Joshi N., Park J., Zhao K., Londo A., Khanal S. (2024). Monitoring harmful algal blooms and water quality using sentinel-3 OLCI satellite imagery with machine learning. Remote Sens..

[13] Zeng F., Song C., Cao Z., Xue K., Lu S., Chen T., Liu K. (2023). Monitoring inland water via Sentinel satellite constellation: A review and perspective. ISPRS J. Photogramm. Remote Sens..

[14] Vivone G., Dalla Mura M., Garzelli A., Restaino R., Scarpa G., Ulfarsson M.O., Alparone L., Chanussot J. (2020). A new benchmark based on recent advances in multispectral pansharpening: Revisiting pansharpening with classical and emerging pansharpening methods. IEEE Geosci. Remote Sens. Mag..

[15] Ghadjati M., Benazza-Benyahia A., Moussaoui A. Satellite image fusion using an iterative IHS-based approach. Proceedings of the 2020 Mediterranean and Middle-East Geoscience and Remote Sensing Symposium (M2GARSS).

[16] Jelének J., Kopačková V., Koucká L., Mišurec J. (2016). Testing a modified PCA-based sharpening approach for image fusion. Remote Sens..

[17] Yilmaz V., Serifoglu Yilmaz C., Güngör O., Shan J. (2020). A genetic algorithm solution to the gram-schmidt image fusion. Int. J. Remote Sens..

[18] Zhong S., Zhang Y., Chen Y., Wu D. (2017). Combining component substitution and multiresolution analysis: A novel generalized BDSD pansharpening algorithm. IEEE J. Sel. Top. Appl. Earth Obs. Remote Sens..

[19] Vivone G., Alparone L., Garzelli A., Lolli S. (2019). Fast reproducible pansharpening based on instrument and acquisition modeling: AWLP revisited. Remote Sens..

[20] Restaino R., Vivone G., Dalla Mura M., Chanussot J., Benediktsson J., Chanussot J., Najman L., Talbot H. (2015). A Pansharpening Algorithm Based on Morphological Filters. Mathematical Morphology and Its Applications to Signal and Image Processing.

[21] Aiazzi B., Alparone L., Baronti S., Garzelli A., Selva M. Advantages of Laplacian pyramids over “à trous” wavelet transforms for pansharpening of multispectral images. Proceedings of the Image and Signal Processing for Remote Sensing XVIII.

[22] Restaino R., Vivone G., Dalla Mura M., Chanussot J. (2016). Fusion of multispectral and panchromatic images based on morphological operators. IEEE Trans. Image Process..

[23] Shi Y., Zhou W., Li W. Pansharpening of multispectral images based on cycle-spinning quincunx lifting transform. Proceedings of the 2019 IEEE International Conference on Signal, Information and Data Processing (ICSIDP).

[24] Khader A., Yang J., Xiao L. (2022). NMF-DuNet: Nonnegative matrix factorization inspired deep unrolling networks for hyperspectral and multispectral image fusion. IEEE J. Sel. Top. Appl. Earth Obs. Remote Sens..

[25] Scarpa G., Vitale S., Cozzolino D. (2018). Target-adaptive CNN-based pansharpening. IEEE Trans. Geosci. Remote Sens..

[26] Yang J., Fu X., Hu Y., Huang Y., Ding X., Paisley J. PanNet: A deep network architecture for pan-sharpening. Proceedings of the IEEE International Conference on Computer Vision.

[27] Fernandez-Beltran R., Fernandez R., Kang J., Pla F. (2023). W-NetPan: Double-U network for inter-sensor self-supervised pan-sharpening. Neurocomputing.

[28] Fernandez R., Fernandez-Beltran R., Kang J., Pla F. (2021). Sentinel-3 super-resolution based on dense multireceptive channel attention. IEEE J. Sel. Top. Appl. Earth Obs. Remote Sens..

[29] Wang Q., Blackburn G.A., Onojeghuo A.O., Dash J., Zhou L., Zhang Y., Atkinson P.M. (2017). Fusion of Landsat 8 OLI and Sentinel-2 MSI data. IEEE Trans. Geosci. Remote Sens..

[30] Shao Z., Cai J., Fu P., Hu L., Liu T. (2019). Deep learning-based fusion of Landsat-8 and Sentinel-2 images for a harmonized surface reflectance product. Remote Sens. Environ..

[31] Jia S., Min Z., Fu X. (2023). Multiscale spatial–spectral transformer network for hyperspectral and multispectral image fusion. Inf. Fusion.

[32] Liu P., Liu J., Xiao L., Zheng Z. (2023). Multiresolution analysis-inspired spatial and spectral details preserved model for variational pansharpening. IEEE Trans. Geosci. Remote Sens..

[33] Yilmaz V. (2023). Adaptive hybrid pansharpening: A novel approach for combining two methods to achieve superior pansharpening performance. Int. J. Remote Sens..

[34] Vivone G., Restaino R., Chanussot J. (2018). Full scale regression-based injection coefficients for panchromatic sharpening. IEEE Trans. Image Process..

[35] Wang P., Yao H., Li C., Zhang G., Leung H. (2021). Multiresolution analysis based on dual-scale regression for pansharpening. IEEE Trans. Geosci. Remote Sens..

[36] Vivone G., Restaino R., Chanussot J. (2017). A regression-based high-pass modulation pansharpening approach. IEEE Trans. Geosci. Remote Sens..

[37] Kumar S., Imen S., Sridharan V.K., Gupta A., McDonald W., Ramirez-Avila J.J., Abdul-Aziz O.I., Talchabhadel R., Gao H., Quinn N.W. (2024). Perceived barriers and advances in integrating earth observations with water resources modeling. Remote Sens. Appl. Soc. Environ..

[38] Jaywant S.A., Arif K.M. (2024). Remote Sensing Techniques for Water Quality Monitoring: A Review. Sensors.

[39] Yang H., Du Y., Zhao H., Chen F. (2022). Water quality Chl-a inversion based on spatio-temporal fusion and convolutional neural network. Remote Sens..

[40] Li J., Dong Z., Chen L., Tang Q., Hao J., Zhang Y. (2025). Multi-Temporal Image Fusion-Based Shallow-Water Bathymetry Inversion Method Using Active and Passive Satellite Remote Sensing Data. Remote Sens..

[41] Shi Y., Tan A., Liu N., Li W., Tao R., Chanussot J. (2023). A pansharpening method based on hybrid-scale estimation of injection gains. IEEE Trans. Geosci. Remote Sens..

[42] Alparone L., Garzelli A., Vivone G. (2017). Intersensor statistical matching for pansharpening: Theoretical issues and practical solutions. IEEE Trans. Geosci. Remote Sens..

[43] Restaino R., Dalla Mura M., Vivone G., Chanussot J. (2016). Context-adaptive pansharpening based on image segmentation. IEEE Trans. Geosci. Remote Sens..

[44] Vivone G. (2019). Robust band-dependent spatial-detail approaches for panchromatic sharpening. IEEE Trans. Geosci. Remote Sens..

[45] Lolli S., Alparone L., Garzelli A., Vivone G. (2017). Haze correction for contrast-based multispectral pansharpening. IEEE Geosci. Remote Sens. Lett..

[46] Palsson F., Ulfarsson M.O., Sveinsson J.R. (2019). Model-based reduced-rank pansharpening. IEEE Geosci. Remote Sens. Lett..

[47] Liu P. (2021). Spatial and spectral anisotropic tensor total variation-driven adaptive pansharpening. IEEE Geosci. Remote Sens. Lett..

[48] Tang Y., Li H., Xie G., Liu P., Li T. (2024). Multi-Frequency Spectral–Spatial Interactive Enhancement Fusion Network for Pan-Sharpening. Electronics.

[49] Maleki S.A., Ghassemian H., Imani M. (2024). Nonreference object-based pansharpening quality assessment. Egypt. J. Remote Sens. Space Sci..

